# The Use of Flow Diverting Stents to Treat Para-Ophthalmic Aneurysms

**DOI:** 10.3389/fneur.2017.00381

**Published:** 2017-08-07

**Authors:** Pervinder Bhogal, Victoria Hellstern, Hansjörg Bäzner, Oliver Ganslandt, Hans Henkes, Marta Aguilar Pérez

**Affiliations:** ^1^Neurocentrum, Klinikum Stuttgart, Stuttgart, Germany

**Keywords:** stents, flow diversion, aneurysm, para-ophthalmic, coiling

## Abstract

**Background and purpose:**

Few publications have dealt exclusively with the use of flow diverter stents for the treatment of para-ophthalmic aneurysms. We sought to determine the efficacy of flow diverting stents (FDSs) to treat aneurysms in this specific location.

**Methods:**

We retrospectively reviewed our database of prospectively collected information for all patients treated with flow diversion for an unruptured saccular para-ophthalmic aneurysm between September 2009 and January 2016. The aneurysm fundus size, neck size, number and type of FDS, complications, and follow-up data were recorded.

**Results:**

We identified 74 patients that matched our inclusion criteria. Of these patients, 18 patients were male (24.3%). The average fundus size was 4.8 mm, 11 aneurysms had previous coil occlusions and 63 were treated solely with flow diversion. At an initial angiographic follow-up (mean avg. 3.2 months), 71.8% of the aneurysms were occluded, and at the last follow-up (mean avg. 31.8 months), 88.9% of aneurysms were occluded. One patient suffered permanent morbidity (1.36%) secondary to interruption of the antiplatelet medication and another died (1.36%) secondary to in-stent thrombosis that was also due to an interruption in the antiplatelet medication.

**Conclusion:**

Treatment of saccular para-ophthalmic aneurysms with FDS is feasible and carries a high degree of technical success with low complication rates and excellent rates of aneurysm exclusion.

## Introduction

The use of flow diverting stents (FDS) has increased in popularity since their introduction into clinical practice. The mechanism of action that results in aneurysm exclusion from the circulation initially involves flow redirection with the subsequent development of a neointimal covering on the surface of the FDS that reconstructs the parent vessel and excludes the aneurysm from the circulation (1). Although early on in their use, there was concern regarding the coverage of side branches and potential ischemia developing, this has largely been shown not to occur with a recent publication by Rangel-Castilla et al. (2) specifically looking at occlusion of covered branches. This study showed variable rates of side branch occlusion but no clinical sequelae seen in any of the patients with occluded branches. Despite the high rates of technical and angiographic success, further more detailed studies are required to determine the exact features that lead to a favorable result such as aneurysm location, presence of arteries derived from the aneurysm sac, and size of the covered branch to name but a few.

In the present study, we present data on all saccular unruptured aneurysms located at the ophthalmic segment of the internal carotid artery (ICA) and treated with FDS in our institution. This is the largest study to date looking at aneurysms arising exclusively from this anatomical location.

## Materials and Methods

### Patient Population

Between September 2009 and January 2016, 74 patients with unruptured aneurysms of the ophthalmic segment (so-called para-ophthalmic aneurysms) were admitted to our institution for endovascular treatment. For each patient, we recorded demographic data, clinical presentation, aneurysm characteristics, therapeutic intervention, immediate angiographic and clinical result, and clinical and radiological follow-up information. The decision to treat is based on a variety of factors and not based on aneurysm size alone. Rather the size, shape, location, presence of other aneurysms, history of subarachnoid hemorrhage, age, and patients wishes are all factored into the decision-making process. The data were entered into our prospectively maintained computer database. Institutional Review Board approval was not required for this retrospective review.

### Classification of the Ophthalmic Segment Aneurysms

The anatomical location of all aneurysms was recorded. Only aneurysms that arose from the ophthalmic segment were included in this study. The ophthalmic segment was classified as the segment of the ICA extending from the distal dural ring to the origin of the posterior communicating artery consistent with the definition of Bouthillier et al. (3).

### Endovascular Treatment

All treatments were performed under general anesthesia. Two commercially available FDS were used: the Pipeline Embolization Device (PED) (Covidien, Irvine, CA, USA) and p64 (phenox, Bochum, Germany). Patient informed consent was obtained before the procedure in all cases. The selection of FDS was dependent upon the operators’ judgment. Selection of the FDS was initially based on availability. Initially only the PED was available, however, after the p64 gained the CE mark, it was also available for use in our department. Furthermore, in our experience, the p64 offers advantages in that it can be completely deployed and resheathed to allow repositioning alongside improved visibility compared to the PED.

All patients received dual antiplatelet therapy (aspirin 100 mg daily and clopidogrel 75 mg daily) for at least 5 days prior to the treatment. The effectiveness of the antiplatelet regime was tested using the Multiplate analyser (Roche, Basel, Switzerland), and since 2016, the VerifyNow test (Accumetrics) was also used. Patients found resistant to clopidogrel received 2 × 90 mg ticagrelor daily. The postprocedural antiplatelet regimen consisted of clopidogrel (or ticagrelor) continued for 12 months following treatment and aspirin continued for life.

Procedures were performed via the right common femoral route using a 6Fr access system as standard and either a Marksman (Medtronic, Dublin, Ireland) catheter or an Excelsior XT27 (Stryker Neurovascular, Kalamazoo, MI, USA) catheter to deploy the FDS. All procedures were performed under heparin anticoagulation with a 5,000 IU bolus dose at the start of the procedure and subsequent 1,000 IU bolus doses every hour to maintain the activated clotting time between 2 and 2.5 times the baseline.

### Procedural Assessment and Follow-up

Patency and flow characteristics within the ophthalmic artery and any cortical branches were assessed angiographically immediately after placement of the FDS and during follow-up. Procedural follow-up (digital subtraction angiography) was performed initially at 3–6 months, again at 9–12 months, and then once per year usually for 3 years or until the aneurysm was excluded from the circulation. Standard angiographic projections were used to assess the patency of the vessels and the aneurysms in addition to angiographic projections that repeated those used during the treatment. Aneurysm occlusion was graded using the Raymond-Roy classification (4) or unchanged (patent).

Neurological examinations were performed to evaluate for potential ischemic or hemorrhagic complications in the postoperative period (<24 h postprocedure) and at each subsequent follow-up. Clinical examination of the visual apparatus included:

- Finger counting.
- Kinetic finger wiggle visual confrontation testing to look for visual field defects.
- Assessment of the extraocular motor nerves.
- Pupillary responses to light, accommodation, and the presence of a relative afferent pupillary defect.

We did not perform fundoscopy as part of the pre- or postoperative assessment nor did we perform retinal photography or fluorescein angiography routinely.

## Results

### Population

In total, we identified 74 patients that met our inclusion criteria (18 males and 56 females). The mean age of the patients was 52.4 years old (range 25–77). All patients had a single aneurysm of the ophthalmic segment of the ICA. Thirty-two were located on the right. The average aneurysm fundus size was 4.8 mm (range 1–16 mm) and the average neck size was 3.5 mm (range 1–10 mm). Fifty-four aneurysms had fundus diameters that were ≤5 mm, 18 were 6–10 mm, and 2 were larger than 10 mm. The baseline demographics and aneurysms characteristics are summarized in Table 1.

**Table 1:** Baseline characteristics of the patients and the aneurysms.

Baseline characteristic	Result
Number	74
Age	52.4 (25–77)
Male	18 (24.3%)
Aneurysm characteristic	
Fundus	4.8 mm (range 1–16)
Neck	3.5 mm (range 1–10)
Coiled	11
Only FDS	63
Treatment	
p64 only	49
PED only	24
p64 + PED	1

### Feasibility

The delivery of the FDS at the initial attempt was possible in all but one case. In this case, the FDS was successfully deployed at the second attempt. In 63 patients, the FDS was used as the sole treatment and in 11 patients additional coiling was required. The PED was used to treat 24 aneurysms, the p64 was used to treat 49 aneurysms, and in one patient both a p64 and a PED were used (Table 1). In total, 52 patients had a single FDS implanted and 22 patients had one than one FDS implanted. One PED was used to treat eight aneurysms, two telescoped PED’s for nine aneurysms, three PED’s for six aneurysms, and four PED’s for one aneurysm. One p64 was used to treat 44 aneurysms, 2 telescoped p64’s for 4 aneurysms and 3 p64’s for 1 aneurysm. One PED and one p64 were used together to treat the aneurysm in one patient.

### Angiographic Follow-up

**Figure 1:**
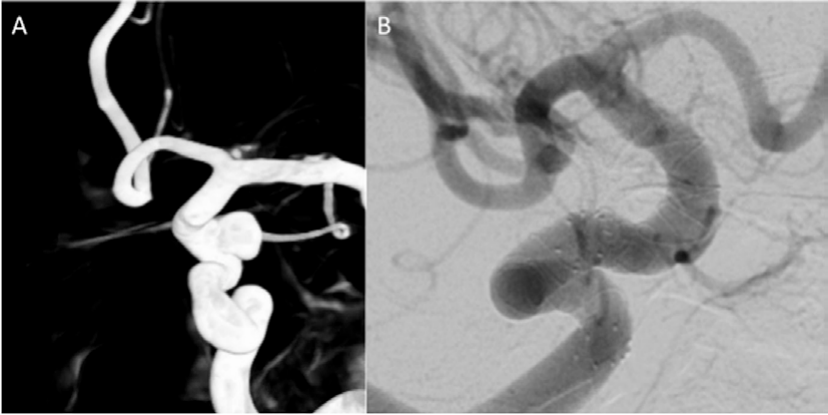
A 6 mm aneurysm can be seen arising from the para-ophthalmic segment of the internal carotid artery (ICA) on the left **(A)**. The aneurysm was treated with two telescoped p64 flow diverting stent (FDS) and the 3-month follow-up angiogram **(B)** shows exclusion of the aneurysm from the circulation.

**Figure 2:**
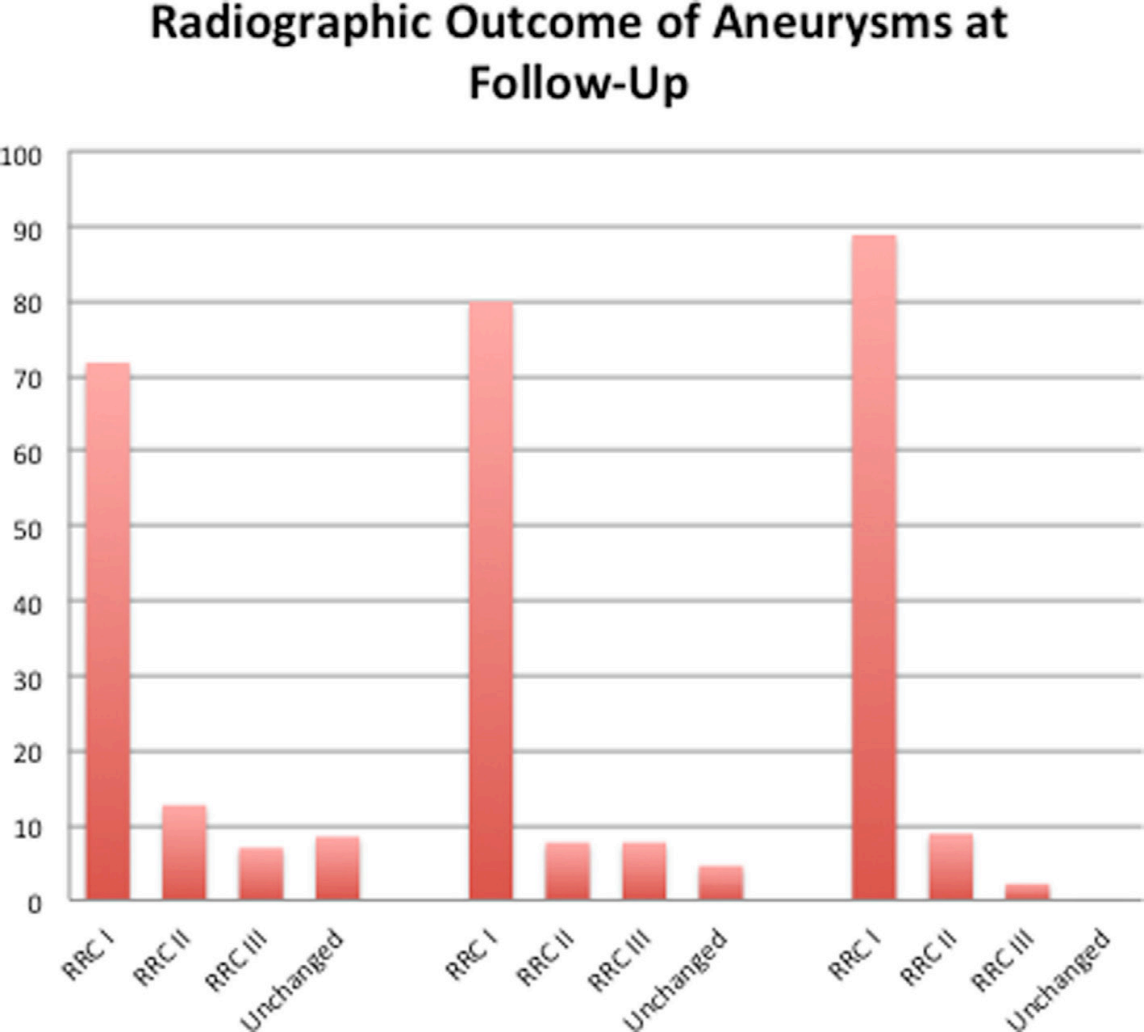
The percentage of aneurysms with Raymond-Roy occlusion grade I, II, or III at initial follow-up (mean 96 days postop, *n* = 71), mid-term follow-up (mean 280 days postop, *n* = 65), and final follow-up (mean 953 days postop, *n* = 45). Increasing occlusion of the aneurysms can be seen over time.

Seventy-one patients have had at least one angiographic follow-up (mean 96 days postop). At this initial angiographic follow-up, 51 aneurysms were completely occluded (71.8%) (Figure 1). Delayed follow-up was available in 45 patients (mean 953 days postop), and in this cohort, 88.9% of aneurysms were completely excluded with neck residuals seen in the majority of the remaining partially excluded aneurysms (8.9%). The results of the angiographic outcome are summarized in Table 2 and in Figure 2. Subgroup analysis showed that of the patients treated with a single FDS (*n* = 48) 30 aneurysms were completely occluded (68.1%); of those treated with more than one FDS (*n* = 22) 20 aneurysms were occluded (90.1%); and those treated with FDS and coils (*n* = 8, all patients had a single FDS) 8 aneurysms were occluded (100%).

**Table 2:** Results of angiographic follow-up.

Angiographic follow-up	Result
Initial (*n* = 71) (mean 96 days postop)	
RRC I	51 (71.8%)
RRC II	9 (12.7%)
RRC III	5 (7%)
Unchanged	6 (8.5%)
Mid-term (*n* = 65) (mean 280 days post-op)	
RRC I	52 (80%)
RRC II	5 (7.7%)
RRC III	5 (7.7%)
Unchanged	3 (4.6%)
Last FU (*n* = 45) (mean 953 days post op)	
RRC I	40 (88.9%)
RRC II	4 (8.9%)
RRC III	1 (2.2%)
Unchanged	0
Ophthalmic artery occlusion	6 (8.1%)

**Figure 3:**
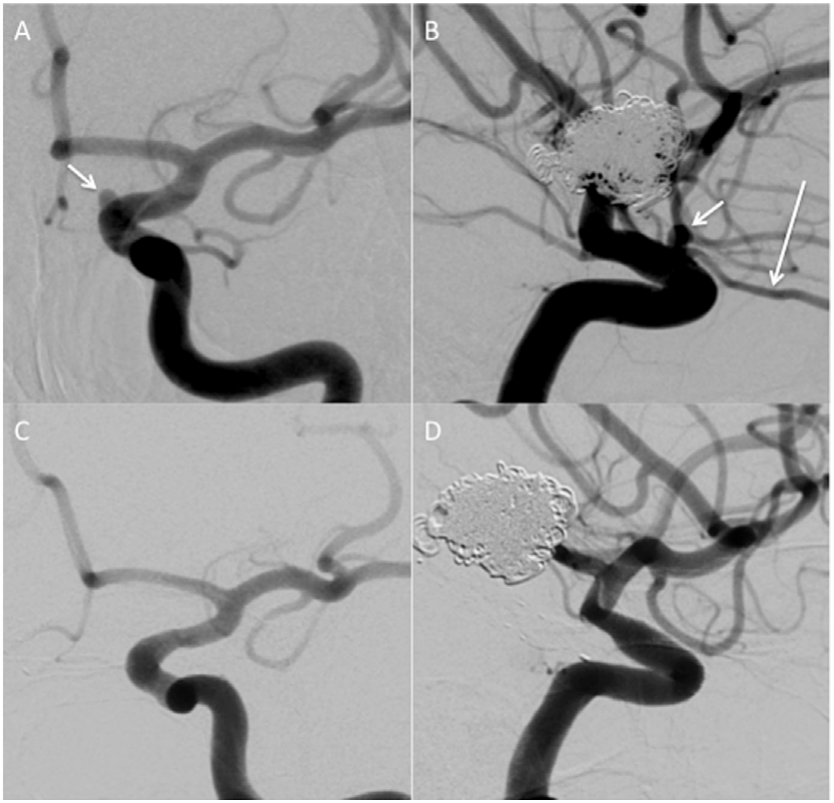
A 3 mm aneurysm arising from the para-ophthalmic segment on the left [**(A,B)**, short white arrows]. Normal filling of the ophthalmic artery is seen [**(B)** long white arrow]. On delayed angiography **(C,D)** the aneurysm is excluded from the circulation and there is no anterograde filling of the ophthalmic artery. The patient did not report any new visual symptoms.

Ophthalmic artery occlusion was seen in six patients (8.1%), none of which resulted in clinical consequences (Figure 3). In four of these occlusion, only a single flow diverter was used.

### Complications

In the periprocedural period (≤24 h), there were two complications. One patient developed a transient leg weakness with a small infarction on the MRI scan. In a second patient, there was an asymptomatic contralateral subarachnoid hemorrhage, discovered on routine postoperative imaging, the cause of which was unknown. In the postprocedural period (>24 h ≤ 30 days), one patient developed an ischemic basal ganglia lesion that was likely secondary to a small embolus since the FDS did not cross the ICA-T in this patient. In another patient, there was a small subarachnoid hemorrhage and in the third the antiplatelet medication was interrupted and the patient suffered from an ischemic stroke (mRS 1 at discharge). In the long-term follow-up period (>30 days), one further patient developed an asymptomatic subarachnoid hemorrhage and one patient suffered a fatal middle cerebral artery infarction secondary to interruption of the antiplatelet medication.

Overall, there was one case of mortality (1.4%) and one case of permanent morbidity (1.4%) that was secondary to antiplatelet medication interruption.

## Discussion

Para-ophthalmic aneurysms account for approximately 5% of all intracranial aneurysms (5) and they represent approximately 0.5% of ruptured aneurysms (6, 7). Although these aneurysms can be treated using a variety of different strategies, the optimal treatment modality is yet to be elucidated.

Surgical treatment of these aneurysms can be complicated by their proximal location, close relationship to the cavernous sinus and the anterior clinoid process as well as the optic nerve and naturally the ophthalmic artery (8–10). All of these factors can make microsurgical clipping of these aneurysms technically challenging. It is also important to realize that mechanical trauma to the optic nerve through retraction is not the only mechanism of injury since heat injury from drilling can also result in injury (9). Since publication of the International Subarachnoid Aneurysm Trial (11) endovascular management of aneurysms has increased with a simultaneous increase in technologies available to the interventional neuroradiologist. The acute occlusion of the ophthalmic artery during either microsurgical clipping or endovascular coiling procedures is to be avoided in order to prevent potential visual complications. While the ophthalmic artery can have a rich collateral supply from branches of the external carotid artery, it is difficult to determine the adequacy of this collateral supply. One method that has been effectively used is balloon test occlusion in the ICA across the origin of the ophthalmic artery prior to coil embolization that may result in acute occlusion of the ophthalmic artery. While BOT can provide useful clinical information (12), it is by no means a guarantee against subsequent visual complications (13). In a recent meta-analysis, Zhu et al. (14) showed in a pooled analysis including 603 aneurysms the total/near total occlusion rate between clipping (89.3%) and stents-assisted coiling (90.7%) showed no significant difference but that both were superior to coiling alone (74.6%). Similarly, there was no significant difference in mortality of patients undergoing treatment with either surgery, coiling or stent assisted coiling, however, higher rates of postprocedural intracranial hemorrhage (ICH) were noted for patients undergoing clipping when compared to coiling and stent assisted coiling (6.4 vs. 2.2 vs. 0%, respectively). Neurological complications were more frequently seen in the clipping group compared to the coiling and stent-assisted coiling group (23 vs. 4.9 vs. 3.9%, respectively) with visual outcomes also showing an advantage over clipping (unfavorable visual outcomes rate for clipping 5.7% for clipping, 1.96% for stent assisted coiling, and 1% for coiling alone). Taking all of this information together it is reasonable to say that the endovascular approach is superior to the microsurgical approach for aneurysms in this location based on the published literature.

The introduction of FDS into clinical practice represented a major leap forward in the endovascular management of aneurysms. Numerous studies have now shown that FDS cause progressive aneurysm occlusion and have a good safety profile (15–33). This phenomenon is seen in our own data with progressive occlusion over time. While many studies did not specify the exact location of treated aneurysms several did include these data (Table 3). The pooled analysis of Zhu et al. (14) also included data regarding the use of FDS and they showed that aneurysm occlusion rates were not significantly different to stent-assisted coiling or clipping (88%), ICH rates were low and similar to other endovascular techniques with an associated low rate of neurological complications (1.75%) and no significant difference in the rate of poor visual outcome between FDS, coiling, and stent-assisted coiling.

**Table 3:** Summary of FDS data regarding treatment of para-ophthalmic aneurysms.

Study	Year of publication	No. of patients	No. of aneurysms	No. of para-ophthalmic aneurysms	Flow diverter							Morbidity	Mortality	Occlusion rate at last FU
					PED	Silk	SURPASS	p64	Tubridge	FRED	Derivo			
Wagner et al. (16)	2012	22	26	11		23						5%	5%	86%
Maimon et al. (17)	2012	28	32	9		31						7.20%	3.60%	83.30%
Mpotsaris et al. (20)	2015	25	28	11		28						8%	4%	59%
Yu et al. (23)	2012	143	178	55	213							3.50%	3.50%	84%
Zanaty et al. (34)	2015	41	44	44	62							2.27%	0.00%	77%
Burrows et al. (35)	2016	44	46	46	NA		NA					4.90%	2.40%	96% (at 3 years)
Wakhloo et al. (27)	2015	165	186	34			165					4.20%	2.40%	75%
De Vries et al. (28)	2013	37	49	9			38					13.50%	0%	94%
Briganti et al. (32)	2016	20	24	10						24		0%	0%	83%
Zhou et al. (36)	2014	28	28	8					33			0.00%	0%	72%
Akgul et al. (37)	2016	24	34	15							27	8.40%	4%	78%
Briganti et al. (31)	2016	40	50	22				NA				2.50%	0%	100%
												5%	2%	82%

Early on their was concern that the coverage of side branches may result in occlusion of these vessels and this phenomenon has certainly been seen. Occlusion of covered branches is the result of the presence of distal collaterals and the suction effect created by lower pressure in these vessels (38). In the presence of collateral flow, a “flow equalization point” may occur that results in the slow flow and occlusion of the proximal vessel proximal to the collaterals (39, 40). In the study of Puffer et al. (41), 21% of patients developed occlusion of the ophthalmic artery and 11% had altered anterograde flow after treatment with the PED, all of which were asymptomatic. Similarly in the recent publication of Burrows et al. (35) occlusion of the ophthalmic artery was seen in 21.6% of patients none of which developed new visual symptoms. In the recent article by Rangel-Castilla et al. (2), the patency of covered branches was reviewed. Of 127 covered arterial branches, of which 76 were ophthalmic arteries, 10.5% were occluded at follow-up (mean angiographic follow-up 10 months). They noted that aside from the number of FDS covering the ophthalmic artery there were no other definite predisposing factors that contributed to vessel occlusion. In our own series, side branch occlusion was seen in six patients (8.1%), which is consistent with the previously reported rates of side-vessel occlusion. While these studies reported no new visual symptoms, it was not made clear how exactly vision was assessed. Small visual defects can be easily underdiagnosed by confrontation testing and the study of Kerr et al. (42) showed that visual field testing using the kinetic finger-wiggle confrontation method was only 39% sensitive for the overall detection of a field defect but only 14.3% sensitive when testing for a mild defect. Therefore, if kinetic finger wiggle, which is the standard bedside clinical assessment performed by most physicians to assess for field defects, was used it is highly likely that even relatively large defects have gone undiagnosed. In an attempt to clarify this, Rouchaud et al. (43) conducted a study with dedicated ophthalmological examination pre and postoperatively with FDS coverage across the ophthalmic artery. The authors classified the aneurysms into four different subtypes:

Type A: the ophthalmic artery originates from the aneurysm sac.
Type B: the ophthalmic artery originates from the neck of the aneurysm.
Type C: the ophthalmic artery originates from the inner curve of the carotid siphon.
Type D: the ophthalmic artery is not involved in the aneurysm but is covered by the FDS.

All patients were given dual antiplatelet therapy for 7 days prior to the intervention and for 3 months after the operation. An ophthalmologist assessed all patients and each patient had, amongst other investigations, retinal photography and retinal fluorescein angiography if any abnormalities were detected. In the follow-up, it was noted that 80% of patients with a type A configuration developed new clinical visual symptoms, all of which were secondary to retinal emboli and 75% of which were permanent. The ophthalmic artery remained patent in all type A cases. The authors believe cause of the new retinal emboli was secondary to thrombosis within the aneurysm sac and then small emboli potentially fragmenting and traveling down the patent ophthalmic artery. Furthermore, the authors also noted a high rate of ischemic optic nerve atrophy associated with a type C configuration. This was believed to be due to the small pore size that inherently occurs along the inner curve of braided stents. Which may then result in poor flow. The authors suggest choosing a FDS stent with slightly larger diameter to avoid excessive compression of the braids. This excellent study provided important information on the potential complications of FDS treatment for para-ophthalmic aneurysms and will no doubt help guide management of these aneurysms based on their anatomical relationships to the ophthalmic artery. However, in certain anatomical dispositions FDS may be the only viable treatment option and from the perspective of other complications such as ICH it is still favorable to surgery and in this case this information serves to guide both the operator and the patient on potential complications.

Our study has several limitations inherent in a retrospective design. We did not analyze the exact anatomical relationship between the ophthalmic artery and the aneurysms in part because we do not routinely perform 3D angiography on our patients as standard. Additionally, we did not carry out detailed ophthalmological and fundoscopic examination pre and postoperatively but rather performed standard bedside clinical assessment to assess for potential visual complications which are likely to have underdiagnosed visual complications as discussed.

## Conclusion

Para-ophthalmic aneurysms can be successfully treated with FDS, and this treatment option carries a high degree of technical success with a low complication rate. Detailed analysis of the anatomical relationship between the ophthalmic artery origin and aneurysm should be performed in order to understand and minimize any potential therapy-related complications.

## Ethics Statement

As this study was retrospective in design and all identifying information removed from images and data, local ethics committee approval was not required. All patients gave informed consent for the procedure. Consent for publication was not required and all identifiable information removed from the manuscript.

## Author Contributions

PB and VH: data collection and analysis and manuscript preparation. OG and HB: review and editing of the manuscript. HH: conceptual design. MP: guarantor, review, and editing.

## Conflict of Interest Statement

MP and PB serve as proctors and consultants for phenox GmbH, with moderate financial compensation. HH is a cofounder and shareholder of phenox GmbH. The other authors have no potential conflict of interest.
